# Focused ultrasound-enhanced nose-to-brain delivery of a therapeutic antibody in a large-animal model

**DOI:** 10.7150/thno.124354

**Published:** 2026-01-01

**Authors:** Siaka Fadera, Cristian Antonio Wieczorek Villas Boas, Yimei Yue, Zhaoning Gu, Jinyun Yuan, Debolina De, Buck E. Rogers, Arash Nazeri, Hong Chen

**Affiliations:** 1 Department of Biomedical Engineering, Washington University in St. Louis, Saint Louis, MO, 63130, USA.; 2 Department of Radiation Oncology, Washington University School of Medicine, St. Louis, MO, 63110, USA.; 3 Mallinckrodt Institute of Radiology, Washington University School of Medicine, St. Louis, MO, 63110, USA.; 4 Department of Neurosurgery, Washington University School of Medicine, St. Louis, MO, 63110, USA.; 5 Division of Neurotechnology, Department of Neurosurgery, Washington University School of Medicine, St. Louis, MO, 63110, USA.

**Keywords:** intranasal brain drug delivery, nose-to-brain delivery, focused ultrasound, microbubble, anti-programmed death-ligand 1 antibody, glymphatic transport

## Abstract

**Background:** The intranasal (IN) route offers a promising noninvasive strategy for central nervous system drug delivery bypassing the blood-brain barrier and reducing systemic exposure. However, its clinical translation is limited by low delivery efficiency and a lack of regional specificity in the brain. Here, we present the first demonstration of focused ultrasound-mediated intranasal delivery (FUSIN) in a large-animal model to address these limitations.

**Methods:** Pigs were used to develop and characterize IN delivery, evaluate systemic exposure in major organs, and assess the feasibility and safety of FUSIN for delivering a therapeutic antibody, anti-programmed death-ligand 1 antibody (aPD-L1). The IN delivery was performed using a catheter-based approach, and successful delivery was confirmed with gadolinium-based contrast agents in combination with magnetic resonance imaging (MRI). Systemic exposure was assessed following IN administration of fluorescently labeled IRDye 800CW-aPD-L1, and its biodistribution was compared with intravenous (IV) injection of IRDye 700CW-aPD-L1. FUSIN delivery was performed by applying focused ultrasound (FUS) to predefined brain targets following IN administration to enhance local antibody accumulation. Delivery outcomes were assessed by *ex vivo* fluorescence imaging, followed by immunofluorescence staining, and safety was evaluated using susceptibility-weighted imaging (SWI).

**Results:** IN delivery targeted the olfactory epithelium region and resulted in significant accumulation of 800CW-aPD-L1 in brain regions associated with the olfactory and trigeminal pathways, while markedly reducing off-target deposition in peripheral organs compared to IV administration. The application of FUS significantly increased local antibody accumulation at the targeted sites compared to contralateral non-sonicated controls. Immunofluorescence imaging revealed FUS-enhanced transport of the antibody from perivascular spaces into the brain interstitial space. SWI detected microhemorrhages under the current FUS parameters, highlighting the need for optimization to ensure safety.

**Conclusion:** This study demonstrates the feasibility of FUSIN for noninvasive, region-specific brain drug delivery with minimized systemic exposure in a large-animal model.

## Introduction

The development of effective therapies for central nervous system diseases is impeded by the blood-brain barrier (BBB), which prevents over 98% of systemically administered drugs from reaching the brain 1. Intranasal (IN) administration provides an unconventional yet promising route for delivering drugs to the brain. This method not only has the promise to circumvent BBB-associated limitations but also to minimize drug accumulation in non-target organs, reducing systemic side effects and enhancing the overall safety of treatments. IN administration has been utilized in preclinical contexts for the delivery of various agents, including therapeutic antibodies, which are crucial in treating neurological diseases, such as brain cancers, Alzheimer's disease, and Parkinson's disease 2-8. However, despite decades of research, clinical translation of IN delivery has been limited by poor delivery efficiency and a lack of regional specificity 9.

Nose-to-brain delivery leverages the unique anatomical and physiological pathways of the nasal cavity, particularly the olfactory and trigeminal nerves 10-12. The olfactory epithelium, located in the upper nasal cavity, contains sensory neurons that extend through the cribriform plate to the olfactory bulb, enabling agents to be transported along these neurons into the brain. Similarly, branches of the trigeminal nerve innervate the nasal mucosa and connect to the brainstem, providing another route for rapid access to the brain. Once enter the brain, the IN-delivered agents are believed to travel along the perivascular spaces (PVS) 12, which are part of the glymphatic system 13. The agents may be propelled through the PVS by arterial pulsation through a mechanism known as the perivascular pumping effect 14.

Despite over 30 years of research on IN brain drug delivery 15, its application remains confined mainly to small animal models, with limited clinical validation. Effective translation to human therapy requires validation in large-animal models with brain sizes, skull thicknesses, and nose-to-brain transport distances comparable to those in humans. However, only a few large-animal studies have been reported on evaluating the nose-to-brain delivery technique 16-19. For example, Thorne *et al.* demonstrated brain delivery of [^125^I]-labeled interferon-β1b in cynomolgus monkeys 16, while Lee *et al.* confirmed BBB bypass and brain accumulation of deuterated-labeled oxytocin in rhesus macaques 17. Sasaki *et al.* emphasized precise olfactory targeting in cynomolgus monkeys for enhanced brain uptake 18. More recently, Smith *et al.* reported superior delivery efficiency of radiolabeled insulin using liquid instillation via catheter compared to aerosol methods in macaques 19. Nevertheless, IN delivery faces significant challenges, such as rapid mucociliary clearance, enzymatic degradation, and poor drug permeability, alongside potential nasal irritation and individual absorption variability 20. Strategies proposed to enhance IN delivery efficiency include the use of mucoadhesive agents, penetration enhancers, and viscosity modifiers, as well as nanocarrier systems such as micelles, polymeric nanoparticles, nanoemulsions, liposomes, solid lipid nanoparticles, and nanostructured lipid carriers 21-24. These pharmaceutical approaches aim to increase the fraction of the administered dose that reaches the brain. However, these strategies primarily focus on overcoming nasal mucosal barriers and do not address the limited ability of IN delivery to achieve region-specific targeting or to significantly enhance penetration into deeper brain structures.

Focused ultrasound-mediated intranasal delivery (FUSIN) was introduced to enhance the delivery efficiency of intranasally administered drugs at a targeted location 25. Focused ultrasound (FUS) is a noninvasive technique that can deliver acoustic energy through the skull to target virtually any location inside the brain. When combined with intravenously injected microbubbles (MBs), FUS induces the expansion and contraction of MBs, exerting a push-pull effect on the adjacent blood vessel wall 26. Recent studies suggest that FUS-induced MB cavitation enhances perivascular transport by causing vessel deformation, a mechanism termed the “MB pumping effect,” which drives fluid transport along PVS and enhances parenchymal penetration of IN-administered drugs 27, 28. Preclinical research has demonstrated that FUSIN can significantly improve the delivery of various agents to distinct brain regions in mice 29-33. For instance, it was reported that FUSIN significantly enhances transgene expression of intranasally administered glial cell line-derived neurotrophic factor (GDNF) DNA nanoparticles specifically in sonicated brain regions 33. This combinatorial FUS-mediated IN delivery approach achieved targeted, localized gene expression while leveraging the non-invasive administration route. Additionally, FUSIN delivery of brain-derived neurotrophic factor (BDNF) was reported to produce neurorestorative effects in a Parkinson's disease mouse model 32. Furthermore, our group found that FUSIN enabled locally enhanced delivery of an anti-programmed cell death-ligand 1 antibody (aPD-L1) to glioma tumors in mice, a promising strategy for enhancing immunotherapy 29.

Despite its great potential, FUSIN has not been evaluated in large-animal models, a critical step for clinical translation. While non-human primates are considered ideal for translational neuroscience, their use is limited by ethical concerns, high costs, and restricted availability. Pigs, by contrast, represent a practical and ethically viable alternative, with brain size and structure that closely approximate those of the human brain. Here, we present the first demonstration of FUSIN in a porcine model, establishing its technical feasibility. As a proof-of-concept, we selected aPD-L1, a clinically relevant immune checkpoint inhibitor, to represent therapeutic antibodies. We developed a catheter-based IN delivery protocol, confirmed precise olfactory epithelium targeting using contrast-enhanced MRI, and demonstrated reduced systemic exposure of IN compared with intravenous (IV) injection. We then developed the experimental procedure for performing FUSIN in pigs and assessed the distribution of IN-administered aPD-L1 in the pig brain. Our results demonstrate that FUSIN increases regional brain antibody accumulation while reducing systemic exposure, highlighting its potential as a noninvasive platform for advancing brain drug delivery.

## Materials and Methods

### Synthesis near-infrared fluorescence dye-labeled aPD-L1

Near-infrared fluorescent labeling of aPD-L1 (IRDye 800CW-aPD-L1) was carried out using our previously established protocol 29. The NHS ester form of IRDye 800CW (LI-COR Biosciences, Lincoln, NE, USA) was used for protein conjugation. Labeling was performed following the manufacturer's instructions, optimized for high-molecular-weight proteins. Briefly, the pH of the aPD-L1 (InVivoMAb, Bio X Cell, Lebanon, NH, USA) solution was adjusted above 7.0 using 1 M potassium phosphate buffer (pH 9.0) to facilitate amine-reactive conjugation. The dye and antibody were mixed at a 2:1 (dye: protein) molar ratio and incubated at room temperature in the dark for at least 2 hours to prevent photobleaching. Following conjugation, excess unbound dye was removed using a 7000 MWCO desalting column (Thermo Scientific, Waltham, MA, USA) pre-equilibrated with 1× PBS. The purified 800CW-aPD-L1 conjugate was stored at 4 °C until further use. In one study, we also prepared 700CW-aPD-L1 following the same protocol to compare the systemic exposure associated with IN and IV delivery, by administering 800CW-aPD-L1 via IN and 700CW-aPD-L1 via IV.

### Animals

All animal procedures were approved by the Institutional Animal Care and Use Committee of Washington University in St. Louis (animal protocol number 25-0283) and conducted in accordance with the National Institutes of Health Guidelines for animal research. Experiments were performed using wild-type Yorkshire white pigs (male, 4 weeks old, ~7 kg; Oak Hill Genetics, Ewing, IL, USA). Animals were initially sedated via intramuscular injection of a ketamine (2 mg/kg), xylazine (2 mg/kg), and telazol (4 mg/kg) cocktail, followed by maintenance of general anesthesia with isoflurane after endotracheal intubation. Before FUS sonication, we applied a hair-removal cream (Nair, Church & Dwight Co., Princeton, NJ, USA) to the cranial region. An IV catheter was placed in the lateral saphenous vein for IV injection.

A total of five studies were performed (**Table 1**). Study 1 aimed to develop an IN administration protocol in pigs and verify that the delivered agent reached the olfactory epithelium using a gadolinium-based contrast agent and MRI. Study 2 aimed to confirm direct brain access of IN-administered aPD-L1. Study 3 aimed to evaluate systemic exposure of IN-administered aPD-L1 in major organs and to compare it with that following IV injection. Study 4 aimed to assess the feasibility of FUSIN delivery of aPD-L1. Study 5 aimed to evaluate safety using susceptibility-weighted imaging (SWI). Detailed experimental procedures for each study are described below.

### Develop an IN-administration protocol and confirm direct brain access

To develop the IN-administration protocol for pigs (Studies 1 and 2,** Table 1**), we modified a previously published protocol for non-human primates 16, 19. Pigs (n=3) were anesthetized as described above and positioned supine with the head slightly elevated and the nose kept at around a 45-degree angle to the table to facilitate nasal delivery. Two flexible catheters (diameter of 1.23 mm, VSI Micro-Introducer Kit, REF 7262V, Teleflex, Vascular Solutions LLC, Minneapolis, MN, USA) were respectively inserted about 7 cm deep into both nostrils. This catheter was specifically chosen because it was thin and flexible, allowing it to be inserted into the deep nasal cavity without causing nosebleeds. Each catheter was then connected to a syringe for the injection of a mixture of gadolinium-based MRI contrast agent, Gadobenate Dimeglumine (Gd-BOPTA; MultiHance, Bracco Diagnostics Inc., Monroe Township, NJ, USA) (0.2 mL/kg) and 800CW-aPD-L1 (1.152 mg/kg), with each nose receiving half of the dose. The gadolinium was used to verify successful IN administration to the nasal epithelium (Study 1), and 800CW-aPD-L1 was used to confirm direct brain access following IN administration (Study 2). Pigs (n=3) without IN administration were included as the control.

Following IN administration, pigs were maintained under anesthesia for 30-60 minutes and then imaged using a 3T MR scanner (Siemens Medical Solutions, Malvern, PA, USA). Anatomic images of the pig head were acquired using two high-resolution 3D MRI sequences, T1-MPRAGE (magnetization-prepared rapid acquisition with gradient echo) and CISS (constructive interference in steady state). A contrast-enhanced T1-weighted (T1-W) SPACE (Sampling Perfection with Application-optimized Contrasts using different flip-angle Evolutions) sequence was acquired to assess the distribution of the contrast agent within the nasal epithelium. The T1-W SPACE sequence provides high-resolution 3D imaging sensitive to gadolinium-based contrast agents. Imaging parameters are summarized in **Table 2**.

After MRI, pigs were sacrificed approximately 2 hours after IN administration. Brains were harvested and fixed with 4% paraformaldehyde. The brains were imaged using the LI-COR Pearl imaging system (LI-COR Biosciences, Lincoln, NE, USA) to detect 800CW-aPD-L1. The mean fluorescence intensity of the whole brain was quantified using LI-COR Image Studio Lite software version 5.2.

### Characterize systemic exposure after IN administration

To assess systemic exposure following IN administration (Study 3,** Table 1**), we employed a paired experimental design using the same animals (n = 4). Each animal received IN administration of 800CW-aPD-L1 and IV injection of 700CW-aPD-L1. The IV route was included as a reference, as it represents the standard delivery method known to produce systemic exposure. By labeling the same antibody with two spectrally distinct near-infrared fluorophores, we achieved direct comparison of the two delivery routes within the same subject. This design enabled precise differentiation and quantification of route-specific distribution while minimizing inter-subject variability.

Pigs were anesthetized, and the animals were positioned supine for IN administration of 800CW-aPD-L1. Immediately following IN administration, 700CW-aPD-L1 was administered intravenously through the IV line. At 5-7 hours after IV injection, the animals were euthanized, and representative tissue samples from peripheral organs, including the heart, lung, liver, stomach, intestine, spleen, and kidney were excised using sterile surgical scalpels and dissecting scissors.

Perfusion was not performed due to the technical complexity of establishing a controlled whole-body perfusion system in large animals. Instead, a post-administration interval of 5-7 hours was selected to balance the competing needs of minimizing residual blood-pool fluorescence and maintaining sufficient tissue signal for quantification. For large-molecule tracers, such as IRDye 800CW-conjugated antibodies, plasma concentrations decline substantially within several hours post-injection due to tissue distribution and hepatic uptake. Sampling at this time point thus enhances tissue-to-blood contrast and provides a practical approximation of organ exposure even without perfusion.

Tissues were imaged using the LI-COR Pearl imaging system under identical acquisition settings for the 700-nm and 800-nm channels. The system provides spectrally distinct detection with minimal crosstalk between channels (700-nm channel: excitation 685 nm, emission 720 nm; 800-nm channel: excitation 785 nm, emission 820 nm). Fluorescence intensity was quantified using LI-COR ImageStudio Lite software by calculating the mean fluorescence intensity across the whole organ. For normalization, the synthesized 800CW-aPD-L1 and 700CW-aPD-L1 solutions were added to conical tubes and imaged under identical conditions, and their mean fluorescence intensities were determined. The relative fluorescence for each organ was calculated as the ratio of the organ's mean fluorescence intensity to the mean fluorescence intensity of the corresponding labeled antibody solution. This ratio provides a semi-quantitative measure of antibody exposure across organs, allowing direct comparison between IN and IV delivery routes.

### FUS setup and FUSIN procedure

The FUS system (Image Guided Therapy, Bordeaux, France) comprised a 15-element annular-ring transducer (Imasonic, Voray-sur-l'Ognon, France; center frequency: 650 kHz, aperture: 65 mm, focal length: 65 mm), a motorized 3D positioning system, and a computer-controlled interface. The FUS transducer was coupled to a water bladder containing room-temperature water that was continuously circulated and degassed through the system's integrated degassing unit. The 3D motor system enabled the precise positioning of the transducer, while the control system regulated the acoustic parameters. Pressure fields generated by the transducer were calibrated using a hydrophone (HGL-0200; Onda Inc., Sunnyvale, USA) in a degassed water tank. The axial and lateral full width at half maximum (FWHM) of the focal zone were 22.0 mm and 3.3 mm, respectively.

For FUSIN delivery, IN administration of 800CW-aPD-L1 was performed 30 minutes before FUS sonication. The drug dose was divided equally between the left and right nostrils and delivered to the pigs in a supine position. After waiting for 30 minutes for drug absorption, the pig was turned to the prone position for the FUS sonication.

For the FUS sonication, a custom-designed head frame was used to stabilize the head and maintain it in a horizontal orientation, following our established protocols 34. The 3D motor was connected to the FUS transducer to align it perpendicular to the skull, achieving an incidence angle of approximately 90°. We adapted our previously established stereotactic-guided FUS brain targeting protocol in mice for use in pigs. In this proof-of-concept study, one side of the hippocampus was selected as a representative deep-brain target. The coordinates of the hippocampus were determined from the pig's anatomic MRI scans. Specifically, the medial-lateral (ML) distance from the hippocampal center to the midline and the dorsal-ventral (DV) distance from the hippocampus to the scalp were measured. The anterior-posterior (AP) distance was measured by determining the distance from the hippocampus to the midpoint of the nose tip. We chose the nose tip as the reference point because it was clearly visible on MRI and easy to identify on the pig. Using the AP and ML coordinates, a reference mark was then drawn on the scalp. The FUS transducer was then positioned using the 3D motor system and aligned with the marked scalp location. The vertical distance from the transducer to the scalp was then set to the focal length minus the measured DV distance, ensuring the focus was aligned with the hippocampus. Ultrasound gel was applied to the skin, and a water container was positioned on the pig's head to provide acoustic coupling with the FUS transducer.

Commercially available MBs (Definity^®^, Lantheus Medical Imaging, North Billerica, MA, USA) were administered intravenously as a bolus injection at a dose of 10 µL/kg, consistent with clinical guidelines. These MBs consist of octafluoropropane gas encapsulated within a lipid shell and have a mean diameter of approximately 1.1-3.3 µm. Immediately after MB injection, one side of the hippocampus was sonicated using ultrasound parameters based on those established in our previous pig studies 34, 35: 0.65 MHz, 3 MPa peak negative pressure as measured in water and 2 MPa measured through a piece of *ex vivo* pig skull, 1 Hz PRF, 10 ms pulse length (duty cycle 1%), and total sonication duration 3 minutes. The contralateral hemisphere served as the non-sonicated control. All FUS parameters are reported in **Table 3**.

### *In vivo* MRI for FUS targeting validation and safety assessment

Following completion of the FUS sonication procedure, the animal was transferred to the MRI scanner for evaluation of BBB permeability and treatment safety. FUS-induced BBB opening (FUS-BBBO) was used to confirm successful FUS targeting. Contrast-enhanced T1-W SPACE MRI was used to assess the BBB permeability. Scans were acquired before and after IV administration of Gd-BOPTA to visualize contrast enhancement at the targeted hippocampal region. Treatment safety was evaluated using SWI, which enables sensitive detection of microhemorrhages or other vascular abnormalities following sonication.

### *Ex vivo* fluorescence imaging for evaluating aPD-L1 delivery outcome

Following MRI scans, pigs were euthanized at 5-7 hours post IN administration, and their brains were carefully extracted and fixed in 10% formalin for a minimum of 48 hours. Brains were then sectioned horizontally from dorsal to ventral to preserve both the FUS-sonicated regions and the corresponding non-sonicated contralateral regions at matching anatomical levels. Uniform 2 mm-thick brain slices were obtained using a custom-designed, 3D-printed brain slicing matrix (A photograph and the 3D printing file of the brain slicing matrix are provided at https://www.protocols.io/private/0F16C5F4AE0911F0AA040A58A9FEAC02).

*Ex vivo* fluorescence imaging was performed using the LI-COR Pearl imaging system to detect 800CW-aPD-L1. All images were acquired under identical imaging conditions, including consistent exposure time across all samples. Fluorescence intensity from each brain slice was quantified using LI-COR Image Studio Lite software version 5.2. For quantitative analysis, regions of interest corresponding to the FUS-treated (FUS+) and contralateral untreated (FUS-) regions were defined. The mean fluorescence intensity within each region of interest was measured, and the values were compared to evaluate signal enhancement in the FUS+ region relative to the FUS- side.

To further investigate the microscopic distribution and underlying mechanisms of IN-delivered 800CW-aPD-L1 following FUSIN treatment, we performed immunofluorescence staining with lectin, a vessel wall marker, and glial fibrillary acidic protein (GFAP), an astrocyte marker. The pig brain slices with enhanced 800CW-aPD-L1 delivery were embedded in a cryomold with Optimal Cutting Temperature medium (SciGen Pte. Ltd., Singapore) and then cut into 20 µm thick brain slices using a cryostat and affixed on glass slides. The slides were stained with anti-GFAP antibody (Abcam, 1:1000) and lectin (Vector Laboratories, 1:1000) to visualize astrocytes and blood vessels, respectively. aPD-L1 was detected using a secondary antibody (Alexa Fluor 594 anti-mouse IgG, Jackson ImmunoResearch, 1:400). All signals were visualized using a fluorescence microscope (BZ-X810, Keyence Corporation, Osaka, Japan).

### Contrast-enhanced MR image analysis

Contrast-enhanced T1-W SPACE MR images obtained from Study 1 and Study 4 were analyzed using a custom MATLAB script, as previously reported 34, 36.

For Study 1, pre- and post-IN contrast T1-W images of the nose were co-registered. The nasal cavity was drawn on the post-IN T1-W image and the pre-IN T1-W image. Contrast enhancement was quantified by thresholding voxel intensities in the post-IN region of interest, retaining only those exceeding three standard deviations above the pre-IN region of interest mean. The contrast-enhanced volume (mm³) was calculated by summing the suprathreshold voxel intensity across all slices.

The same procedure was followed for quantifying FUS- vs FUS+ contrast enhancement in Study 4. Regions of interest covering the hippocampus were drawn on the FUS-treated (FUS+) and contralateral non-FUS-treated brain regions (FUS-). Voxels in the FUS+ region of interest with intensities greater than three standard deviations above the mean intensity of the FUS- region of interest were identified for each MRI slice. The contrast-enhancement volume was calculated by summing the identified voxels across the entire brain.

### Statistical analysis

Statistical analysis was performed using GraphPad Prism (Version 10, Boston, MA, USA). Representative data were presented as mean values ± standard deviation. Where appropriate, the statistical significance difference between the experimental groups was analyzed using the unpaired and paired two-tailed Student's t-test, unless otherwise specified.

## Results

### IN-administered MRI contrast agents reached the olfactory epithelium

**Figure 1:**
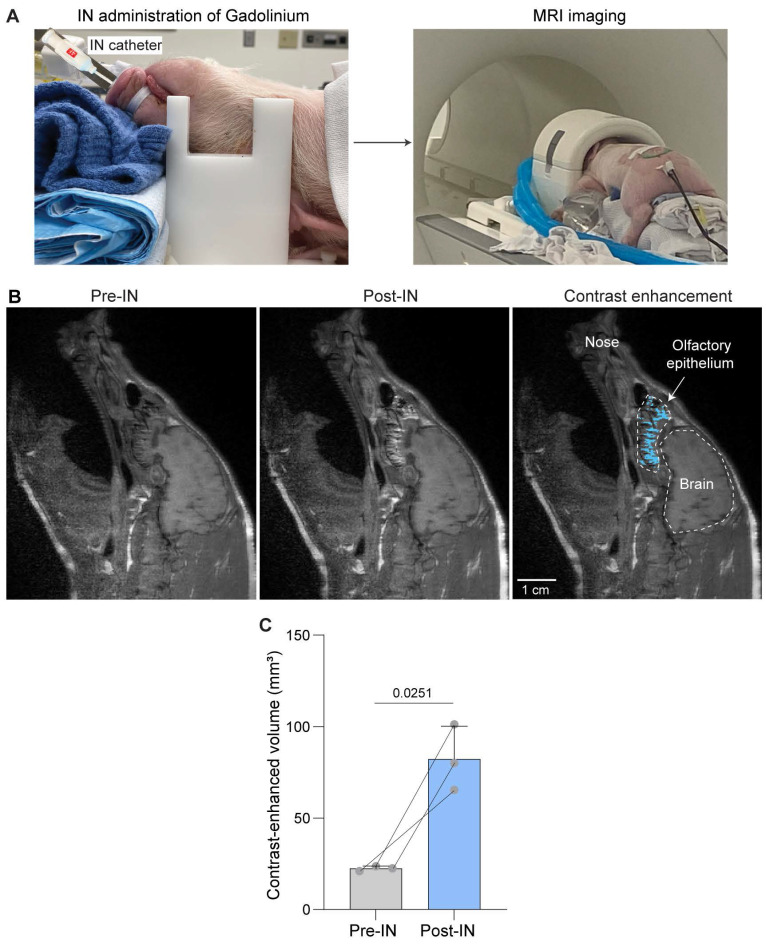
** MRI confirmation of Gd-BOPTA delivery to the olfactory epithelium following IN administration.** (A) Schematic illustration of IN administration of Gd-BOPTA into the nasal cavity and subsequent MRI acquisition. (B) Representative sagittal MRI images acquired before and after IN administration, showing contrast enhancement in the olfactory region (highlighted in blue). (C) Quantification of Gd-BOPTA signal enhancement along the olfactory epithelium, demonstrating significant uptake in the olfactory epithelium post-IN delivery. Bar plots show the mean and standard deviation. Individual animal data points are connected by lines to indicate paired measurements. Statistical significance was assessed using a paired two-tailed Student's t-test.

We used the flexible catheter for the IN administration to the olfactory epithelium (**Figure 1A**). To verify precise targeting of the olfactory epithelium, we administered MRI contrast agent Gd-BOPTA. As shown in **Figure 1B**, contrast-enhanced MRI revealed clear contrast enhancement in the nasal epithelium regions above the cribriform plate post-IN. Quantification of MRI images found that the contrast-enhanced volume in the olfactory epithelium increased by 3.7-fold (22.4 ± 1.3 mm^3^ for pre-IN and 82.2 ± 18.1 mm^3^ for post-IN, **Figure 1C**), confirming the effective targeting of the olfactory epithelium using the developed IN administration approach.

### IN-administered 800CW-aPD-L1 reached the brain

**Figure 2:**
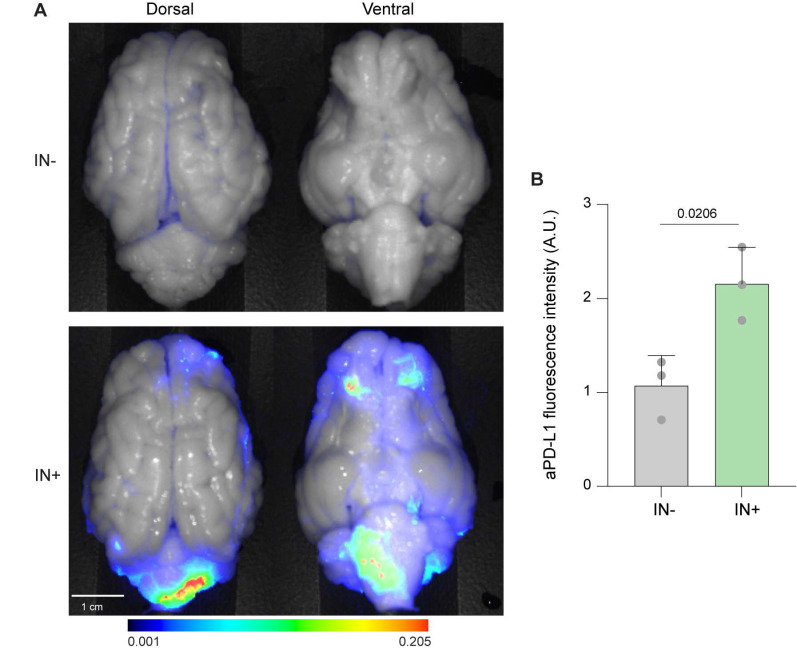
** Fluorescence imaging of 800CW-aPD-L1 following IN administration.** (A) *Ex vivo* ventral and dorsal whole-brain fluorescence images of pig brains following IN administration (IN+) or no IN (IN-) administration of 800CW-aPD-L1. Fluorescence signals are overlaid on corresponding bright-field images. Stronger fluorescence was observed in the IN+ brains, particularly in the olfactory bulb and brainstem regions, consistent with known entry routes along the olfactory and trigeminal pathways. (B) Quantification of aPD-L1 fluorescence intensity from whole-brain images. Bar plots represent mean ± standard deviation. Each data point represents an individual animal. Statistical significance was assessed using an unpaired two-tailed Student's t-test.

Using the established IN administration protocol, we performed IN delivery of 800CW-aPD-L1 in pigs. Fluorescence signals were detected in the *ex vivo* brain post-IN administration. Strong fluorescence signals were observed in ventral brain regions around the olfactory and brainstem regions, consistent with uptake via nasal-associated pathways (**Figure 2A**). This pattern aligns with the anatomical proximity of the olfactory and trigeminal nerves, which provide direct access to the brain. The olfactory route offers entry into the olfactory region, while the trigeminal pathway facilitates delivery to the brainstem. These findings confirmed direct access to the brain from the IN administration.

### IN administration minimized systemic exposure

**Figure 3:**
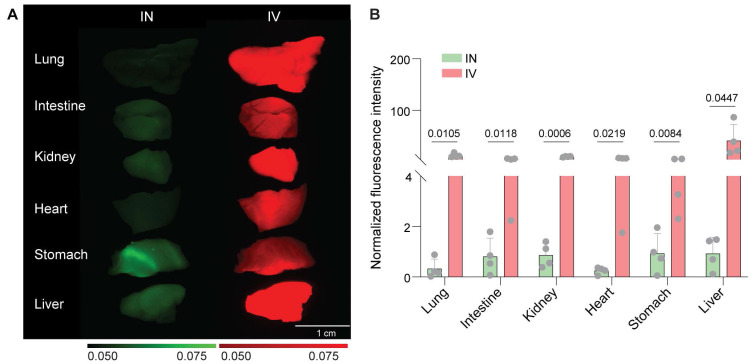
** IN delivery reduced systemic exposure compared to IV injection.** (A) Representative *ex vivo* fluorescence images of major organs (lung, intestine, kidney, heart, stomach, and liver) following IN delivery of 800CW-aPD-L1 or IV delivery of 700CW-aPD-L1. (B) Quantification of mean fluorescence intensity (MFI) across organs, normalized to the mean fluorescence intensity of the administered aPD-L1. The data illustrate distinct biodistribution profiles for each route, with IN administration showing significantly lower fluorescence in non-target organs compared with IV administration. Bar plots show the mean and standard deviation. Each data point corresponds to an individual animal. Statistical significance was assessed using an unpaired one-tailed Student's t-test.

The comparison between IN and IV administration of aPD-L1 revealed a clear advantage of the nasal route in reducing systemic exposure. Fluorescence images reveal that the IV route results in the widespread accumulation of the drug in peripheral organs, including the heart, lung, liver, stomach, intestine, and kidney, indicating significant systemic exposure, as shown in **Figure 3**. In contrast, IN administration resulted in lower fluorescence in these organs, confirming minimized off-target distribution.

### FUSIN delivery of 800CW-aPD-L1 was feasible

**Figure 4:**
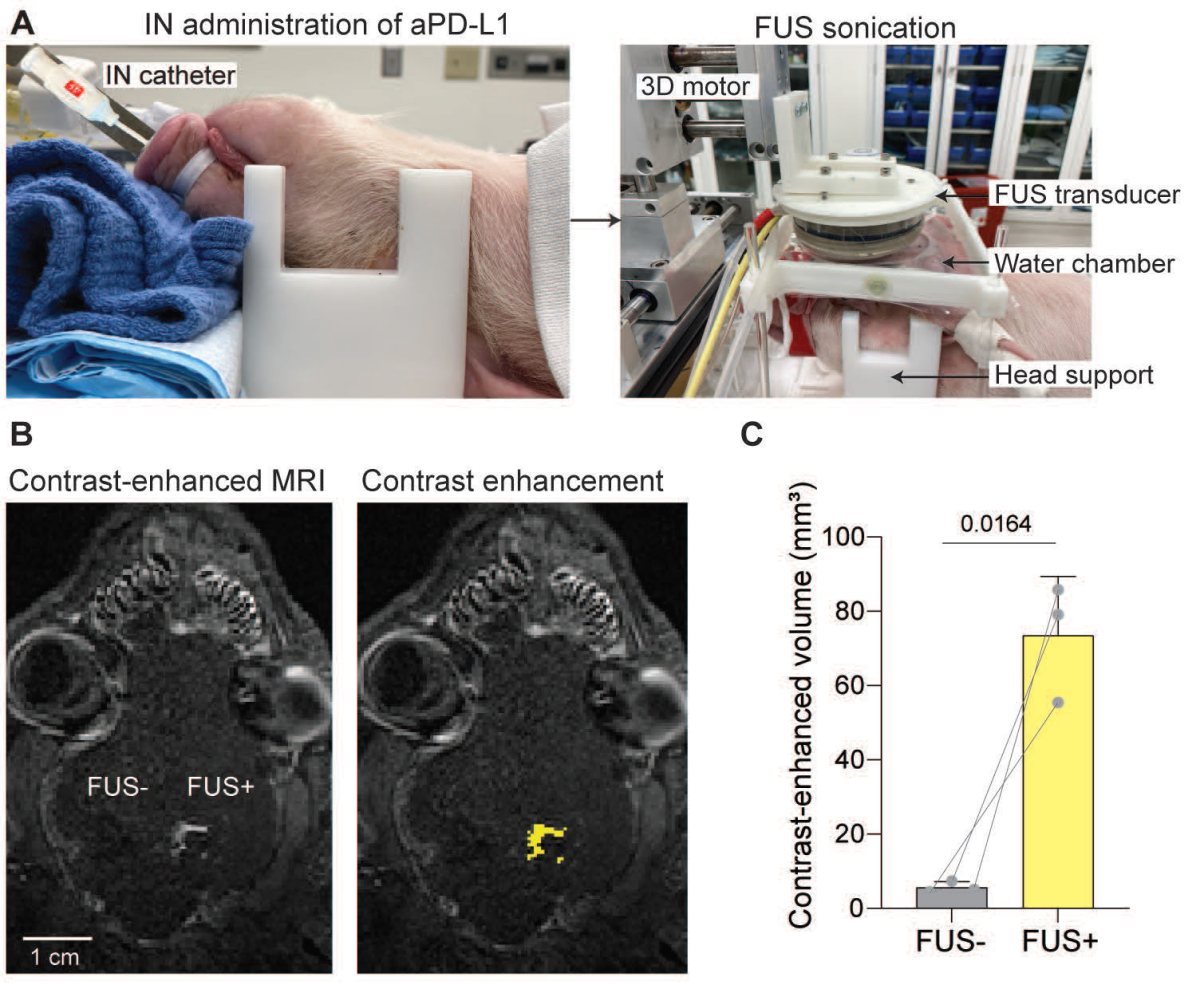
** Contrast-enhanced MRI confirmed localized brain targeting by FUS.** (A) Schematic of FUSIN experimental setup. Left: IN administration of aPD-L1 using a catheter. Right: FUS sonication setup showing the pig positioned with its head secured beneath a water-coupled FUS transducer mounted on a 3D motor system. (B) Representative horizontal T1-weighted contrast-enhanced MRI images showing localized FUS targeting (FUS+) compared with the non-sonicated control hemisphere (FUS-). (C) Quantification of contrast-enhanced volume in FUS- versus FUS+ regions, showing a significant increase in contrast enhancement following FUS. Bar plots show the mean and standard deviation. Individual animal data points are connected by lines to indicate paired measurements. Statistical significance was assessed using a paired two-tailed Student's t-test.

We developed the experimental protocol for performing FUS combined with MB treatment after IN administration (**Figure 4A**). The FUS system could target the desired brain region, as verified by Gd-BOPTA extravasation after FUS sonication (**Figure 4B**). Quantitative volumetric analysis revealed a significant contrast enhancement in the FUS-targeted region compared to the contralateral non-targeted control region (**Figure 4C**).

**Figure 5:**
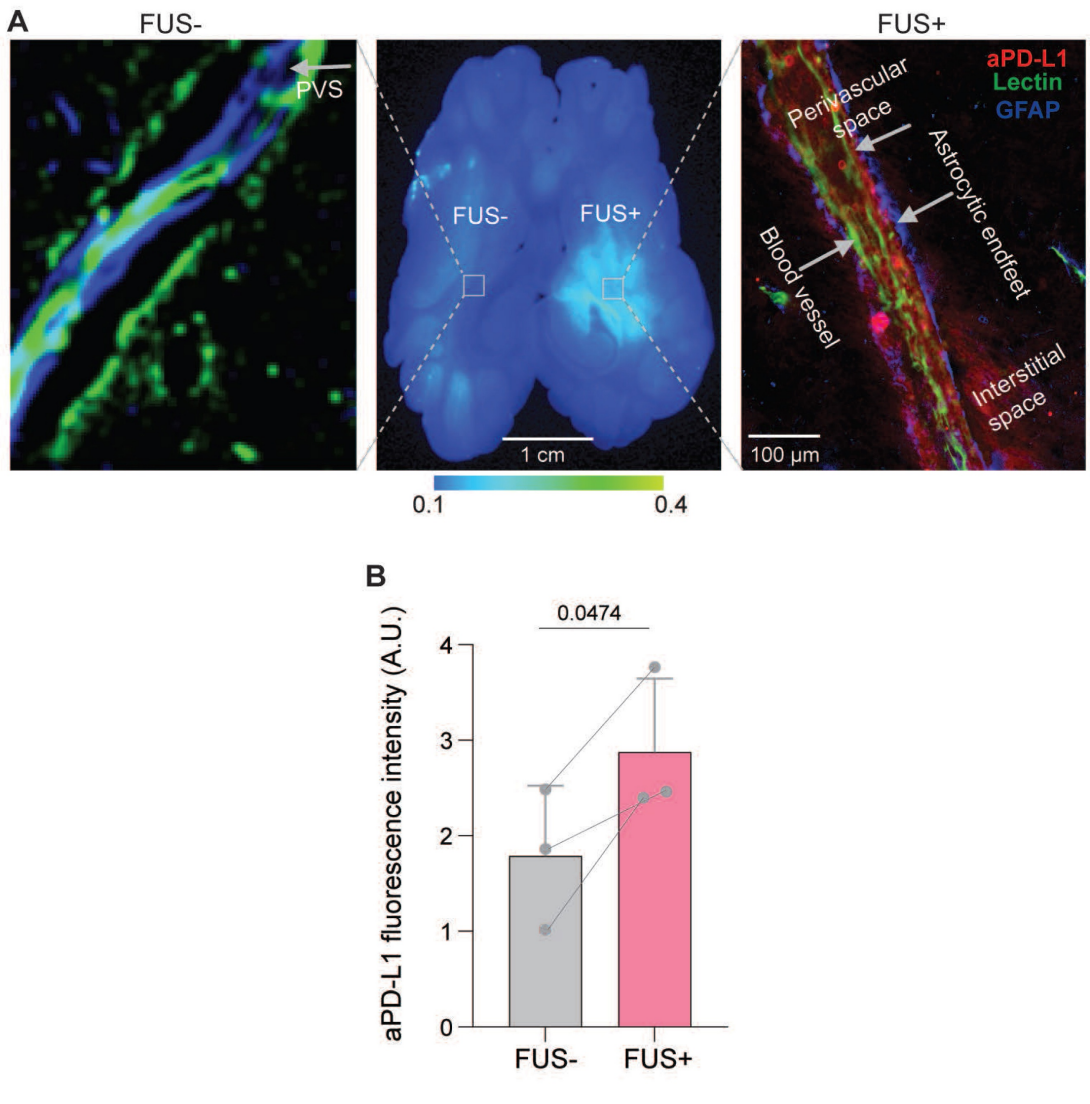
** FUSIN enhanced the accumulation of aPD-L1 at the FUS-targeted brain location.** (A) Representative images showing the distribution of fluorescently labeled aPD-L1 in the brain with and without FUS. Left: High-resolution microscopic image from a FUS- region showing minimal aPD-L1 (red) signal. Middle: Whole-brain slice fluorescence image demonstrating higher accumulation of aPD-L1 in the FUS+ hemisphere compared with the FUS- side. Right: High-resolution microscopic image from a FUS+ region revealing accumulation of aPD-L1 (red) in the perivascular space (PVS) and interstitial space. (B) Quantification of aPD-L1 fluorescence intensity in FUS- and FUS+ regions showing a significant increase in aPD-L1 delivery following FUS. Bar plots show the mean and standard deviation. Individual animal data points are connected by lines to indicate paired measurements. Statistical significance was assessed using a paired two-tailed Student's t-test.

**Figure 5A** shows a horizontal brain slice comparing 800CW*-*aPD-L1 accumulation between FUS-targeted and non-targeted contralateral brain regions. Notably, greater 800CW-aPD-L1 accumulation was observed in the FUS-targeted region. Quantitative analysis (**Figure 5B**) revealed an average 1.6-fold increase in fluorescence intensity in the FUS-targeted area compared to the contralateral non-targeted region.

In the FUS-targeted brain region (**Figure 5A**), aPD-L1 was observed in the PVS between the vessel walls and astrocytic endfeet and the interstitial space (fluorescence was observed extending from the PVS into the interstitial space by a maximum of ~100 μm). This was not observed in the contralateral non-targeted region. These findings highlight the potential of FUS to enhance IN drug delivery to the brain tissue by promoting perivascular transport and penetration through the PVS to the interstitial space.

### Safety assessment of FUS treatment using MRI

**Figure 6:**
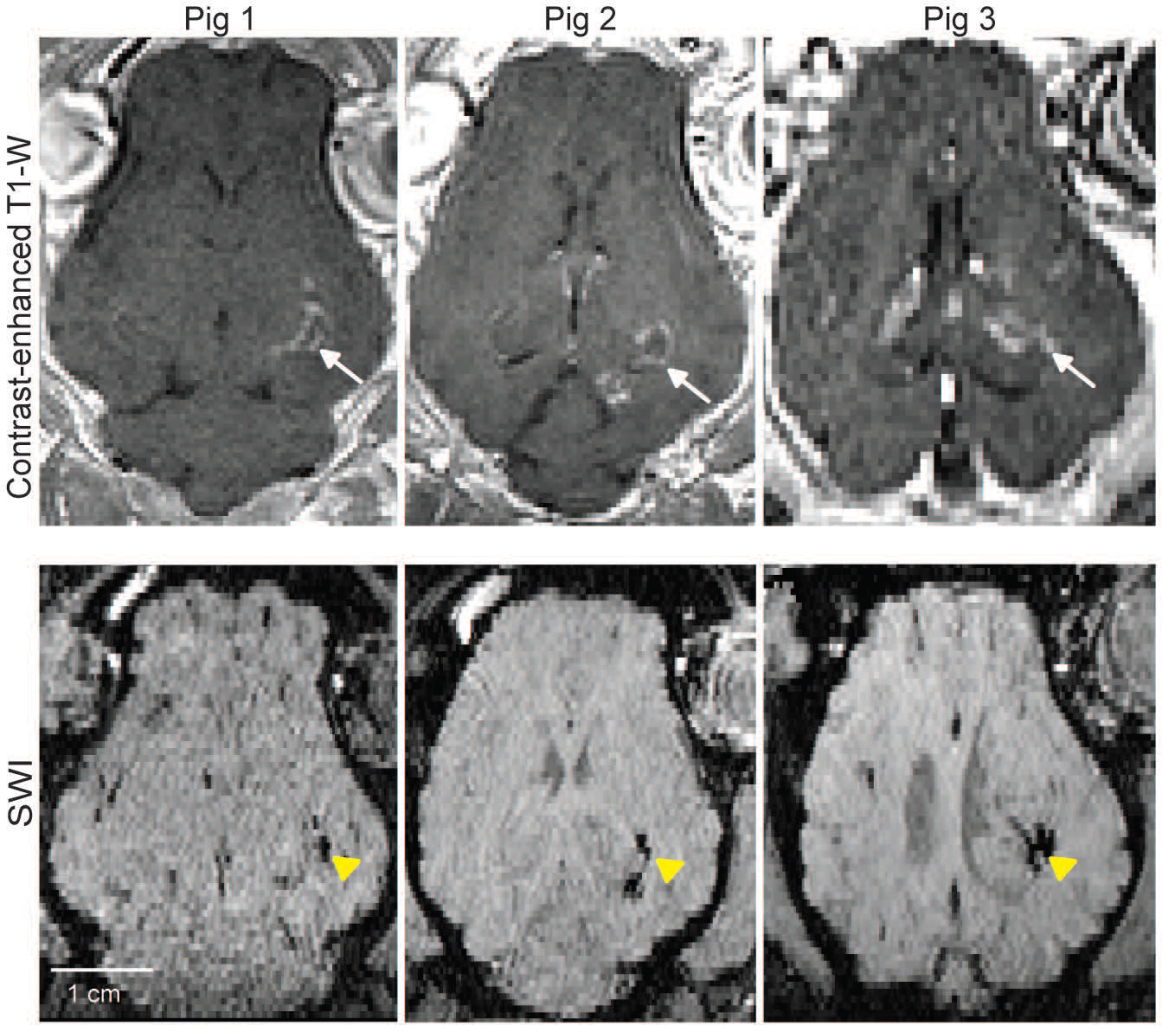
** MRI safety evaluation.** Representative horizontal MRI images of pig brains. Top row: Contrast-enhanced T1-W images show localized signal enhancement at the targeted brain regions (white arrows), confirming successful FUS targeting. Bottom row: Corresponding SWI reveal microhemorrhage in the targeted regions (yellow arrowheads).

**Figure 6** shows representative contrast-enhanced T1-W (top row) and SWI (bottom row) images following FUS treatment. The FUS treatment resulted in distinct microhemorrhages, visible as hypointense regions on SWI (yellow arrowheads), indicative of vascular injury. Consequently, this highlights the critical need to optimize FUS parameters to ensure safety without compromising therapeutic efficacy.

## Discussion

This study presents the first demonstration of FUSIN in a large-animal model, establishing its potential as a noninvasive, spatially targeted strategy for brain drug delivery. Previous work in mice demonstrated that combining IN administration with MB-enhanced FUS can significantly increase drug penetration into targeted brain regions by promoting perivascular transport and parenchymal distribution 28. Our findings extend this concept to the porcine brain, a species with neuroanatomical features, including brain size, skull thickness, and nose-to-brain transport distance, more comparable to humans than rodents, thus bridging an important translational gap.

A critical first step for successful nose-to-brain delivery is precise deposition of the therapeutic agent in the olfactory epithelium, enabling transport along the olfactory and trigeminal pathways. Prior large-animal studies have shown that direct instillation to the olfactory region improves delivery efficiency. For example, Thorne *et al.* used flexible plastic tubing to administer [^125^I]-labeled interferon-β1b to cynomolgus monkeys, while Smith *et al.* reported that catheter-based delivery of insulin liquid outperformed aerosolized methods in non-human primates when assessed by PET imaging 16, 19. In our study, contrast-enhanced MRI confirmed that catheter-based liquid administration in pigs successfully targeted the olfactory epithelium, validating the feasibility of this approach in a non-primate large-animal model. This is particularly important given the greater anatomical complexity of the porcine nasal cavity compared with rodents, which may otherwise hinder efficient olfactory deposition.

A major advantage of IN administration is its ability to minimize systemic exposure, thereby reducing off-target exposure. Consistent with prior reports in rodents 25, 38, we observed that IN administration substantially reduced systemic exposure compared with IV delivery in pigs. This is a key advantage for brain-targeted biologics, such as immune checkpoint inhibitors, where peripheral immune activation can lead to systemic toxicity. Our fluorescence imaging revealed markedly lower aPD-L1 accumulation in major peripheral organs following IN administration, confirming that minimizing systemic circulation is feasible in a large-animal setting.

The principal advance of this work is the demonstration that FUS substantially enhances the accumulation of intranasally delivered antibodies in the targeted brain region in a large-animal model. In mice, FUSIN has been shown to enhance the delivery of nanoparticles, viral vectors, and proteins specifically to sonicated regions 29-33. Here, we observed a 1.6-fold increase in aPD-L1 accumulation in FUS-targeted areas relative to contralateral controls. Although this enhancement was smaller than the ~4-fold increase observed in our prior glioma-bearing mouse study 29, our findings confirm that FUSIN can be implemented in a species with human-relevant neuroanatomy. Immunofluorescence further revealed antibody localization within both PVS and interstitial space, consistent with the proposed “MB pumping effect” in which MB oscillations deform vessel walls, facilitating convective transport along the PVS and improving penetration into interstitial space. Our data provide the first evidence that this mechanism can be harnessed in a large-animal model to improve targeted delivery of large biologics.

FUSIN and FUS-BBBO utilize the same FUS sonication technique, with the main procedural difference being the drug administration route. We observed an average 1.6-fold increase in aPD-L1 accumulation in FUSIN-targeted regions relative to contralateral controls. This enhancement was slightly lower than, but comparable to, the 2.1-fold increase achieved in our prior study that delivered the same aPD-L1 to the brain using FUS-BBBO in combination with IV injection 34. The FUS parameters and the aPD-L1 drug doses were identical in these two studies. A key similarity lies in the final outcome: both FUS techniques successfully enhanced the localized delivery of antibodies to the brain parenchyma in a large-animal model. The differences, however, are substantial. Mechanistically, FUS-BBBO relies on transient BBB opening to facilitate the passive extravasation of aPD-L1, which was systemically administered after FUS sonication. Conversely, FUSIN utilizes intravascular MB oscillations to actively drive convective transport along the PVS, a process enabled by administering the antibody before FUS sonication. The drug delivery enhancement ratios obtained by FUSIN and FUS-BBBO are comparable; however, a conclusion cannot be drawn in terms of which technique has higher drug delivery efficiency for several reasons. First, the experimental conditions were not identical. Our previous FUS-BBBO studies targeted the cortex, whereas FUSIN targeted the hippocampus. Furthermore, the animal sacrifice timeline was extended to at least twice as long post-IN administration in the present FUSIN study. Second, this is the first study to investigate the feasibility of FUSIN, with future studies needed to optimize the treatment parameters to maximize the delivery efficiency, while FUS-BBBO is a relatively mature technique with more than two decades of research on optimizing it. Nevertheless, both methods achieve the primary goal of localized brain delivery. However, FUSIN provides a complementary strategy that reduces systemic toxicity.

Our observation of aPD-L1 distribution within the PVS suggests that glymphatic transport along these pathways may play a critical role in its brain delivery. It is important to note that IN administration, particularly when facilitated by FUS, can engage multiple routes of entry into the brain. These include, but are not limited to, the para-neuronal olfactory pathway along the paraneural space 37, the trans-neuronal pathway 37, entry into the cerebrospinal fluid followed by distribution through the glymphatic system 38, and systemic absorption with subsequent FUS-BBBO. Future studies are warranted to delineate the relative contributions and underlying mechanisms of these pathways in FUSIN delivery.

Microhemorrhages were observed in the FUS-targeted regions, consistent with previous reports indicating that such effects are typically transient 39, 40. Notably, these microhemorrhages were localized to the sonicated areas, suggesting that FUS targeting induces confined tissue damage with minimal off-target parenchymal effects. Prior studies have documented similar FUS-induced tissue damage, with recovery occurring within 4 days in mice and 1-2 months in humans 41-43. Supporting this, Lipsman *et al.* reported comparable transient damage in their clinical MRI-guided FUS-BBBO study, where microhemorrhages resolved within 24 hours 40.

The primary cause of vascular damage in this study was the relatively high acoustic pressures used. Future studies will investigate lower acoustic pressures to mitigate vascular injury while maintaining effective drug delivery enhancement. Another potential contributing factor was the use of a bolus MB injection, which can produce transiently high microbubble concentrations within the FUS focus immediately after administration. Employing a continuous infusion via an infusion pump could help maintain a more stable microbubble concentration during sonication, thereby reducing the risk of transient cavitation events and vascular rupture. A further limitation of our current setup is the lack of skull aberration correction. Accurate transcranial targeting is difficult to achieve without compensating for phase distortions introduced by the skull. Future studies should consider using a phased-array transducer with a larger number of elements (e.g., 128 or 256) to enable aberration correction and improve focal precision. Another important experimental parameter to optimize is the time delay between IN administration and FUS sonication. Our previous FUSIN study in mice suggested that delivery efficiency is maximized when FUS is applied at the time point when the IN-administered agent reaches its peak concentration in the targeted brain region 31. Future studies should characterize the pharmacokinetic profile of the IN-delivered agent within the brain and optimize FUSIN delivery by determining the optimal delay time.

While this study provides foundational insights into FUSIN delivery, some limitations warrant further consideration. First, although multimodal imaging and histology were used to assess delivery efficiency and localization, therapeutic efficacy was not evaluated in a disease model; thus, the functional impact of aPD-L1 delivery remains unknown. Second, while pigs provide superior translational relevance compared to rodents, interspecies differences in nasal anatomy influence delivery dynamics when extrapolating to humans. Third, aPD-L1 distribution was assessed at a single time point; radiolabeling aPD-L1 in future studies would allow for spatiotemporal mapping of its distribution in the brain and body via PET imaging. Fourth, while *ex vivo* fluorescence imaging provided valuable spatial information on antibody distribution within the brain, it was inherently semi-quantitative. Complementary quantitative approaches, such as enzyme-linked immunosorbent assay (ELISA) or mass spectrometry, would enable the absolute quantification of antibody concentrations in both targeted and off-target regions. Future studies will incorporate these assays to validate fluorescence-based measurements and provide a more comprehensive understanding of antibody biodistribution following FUSIN treatment. Finally, safety evaluation in this study was limited to acute imaging outcomes. Although no extensive tissue damage was observed, the presence of localized microhemorrhages underscores the need for more comprehensive safety assessments. Future studies will focus on optimizing ultrasound parameters to minimize vascular perturbation while maintaining delivery efficacy. In addition, systematic safety evaluations over extended time points, including histopathological analysis, longitudinal imaging, behavioral testing, and repeated dosing paradigms, will be essential to fully characterize both the safety profile and therapeutic potential of FUSIN prior to clinical translation.

## Conclusion

This study demonstrates the feasibility of FUSIN for the delivery of aPD-L1 in a porcine model. MRI confirmed accurate IN targeting of the olfactory epithelium. Compared to IV injection, IN administration significantly reduced systemic exposure, highlighting its potential for minimizing off-target effects. Importantly, FUSIN enabled localized enhancement of aPD-L1 delivery to specific brain regions, with antibody accumulation observed in both perivascular and interstitial spaces. These findings establish FUSIN as a promising approach for region-specific brain drug delivery and support further development and safety optimization for future therapeutic applications in humans.

## Figures and Tables

**Table 1:** Summary of studies.

	Animal #	Study #	Study goal	Readout
IN	n=6	1	Develop IN administration protocol in pigs and verify successful administration to the olfactory epithelium	Contrast-enhanced MRI
		2	Confirm direct access to the brain following IN administration of aPD-L1	Fluorescence imaging
	n=4	3	Characterize systemic exposure	Fluorescence imaging
FUSIN	n=3	4	Evaluate the feasibility of FUSIN delivery of aPD-L1	Fluorescence imaging
		5	Characterize the safety of FUSIN	SWI MRI

**Table 2:** MRI acquisition parameters.

Sequences	TR/TE	Slice thickness	Matrix size	In-plane resolution
3D T1-MPRAGE	2300/3.4 ms	0.7 mm	352 × 352	0.7 × 0.7 mm^2^
3D CISS	8/3.5 ms	0.7 mm	512 × 352	0.3 × 0.3 mm^2^
3D T1-W SPACE	700/9.6 ms	0.7 mm	320 × 320	0.7 × 0.7 mm^2^
3D SWI	29/21 ms	1.0 mm	448 × 213	0.4 × 0.4 mm²

TR: repetition time, TE: echo time, MPRAGE: magnetization-prepared rapid acquisition with gradient echo, CISS: constructive interference in steady state, T1-W SPACE: T1-weighted sampling perfection with application-optimized contrasts using different flip-angle evolution, SWI: susceptibility-weighted imaging

**Table 3:** FUS sonication parameters

Category	Parameter	Value
Transducer & System	Manufacturer/Model	Imasonic, 15-element annular-ring transducer
	Center frequency	650 kHz
	Aperture diameter	65 mm
	Focal length	65 mm
	Drive system	Image Guided Therapy (IGT)
Calibration & Beam Profile	Hydrophone calibration	HGL-0200, Onda
	Peak negative pressure in water	3 MPa
	Axial FWHM	22.0 mm
	Lateral FWHM	3.3 mm
Exposure Parameters	Pulse repetition frequency (PRF)	1 Hz
	Pulse length	10 ms
	Sonication duration	3 minutes
Targeting & Coupling	Targeted brain region	Hippocampus
	Animal positioning	Prone position
	Coupling medium	Degassed ultrasound gel + water chamber
	Incident angle	~90° to skull
Microbubble	Brand	Definity^®^
	Dose	10 µL/kg
	Injection method	Bolus

## Abbreviations

FUS: focused ultrasound
MB: microbubble
IN: intranasal
FUSIN: focused ultrasound-mediated intranasal delivery
PVS: perivascular space
BBB: blood-brain barrier
aPD-L1: anti-programmed cell death-ligand 1 antibody
IV: intravenous
MRI: magnetic resonance imaging
T1-W: T1-weighted
FUS-BBBO: FUS-induced blood-brain barrier opening
